# More income, less depression? Revisiting the nonlinear and heterogeneous relationship between income and mental health

**DOI:** 10.3389/fpsyg.2022.1016286

**Published:** 2022-12-14

**Authors:** Chao Li, Guangjie Ning, Lin Wang, Feier Chen

**Affiliations:** ^1^Research Unit of Public Health and Health Economics, Research Center of Labor Economics, Business School, Shandong University, Weihai, China; ^2^Glorious Sun School of Business and Management, Donghua University, Shanghai, China

**Keywords:** income, depression, mental health, U-shaped effect, heterogeneity

## Abstract

This paper uses a large-scale nationally representative dataset to examine the nonlinear effect of income on mental health. To investigate their causal relationship, the exogenous impact of automation on income is utilized as the instrument variable (IV). In addition, to explore their nonlinear relationship, both income and its quadratic term are included in regressions. It is found that the impact of income on mental health is U-shaped rather than linear. The turning point (7.698) of this nonlinear relation is near the midpoint of the income interval ([0, 16.113]). This suggests that depression declines as income increases at the lower-income level. However, beyond middle income, further increases in income take pronounced mental health costs, leading to a positive relationship between the two factors. We further exclude the possibility of more complex nonlinear relationships by testing higher order terms of income. In addition, robustness checks, using other instrument variables and mental health indicators, different IV models and placebo analysis, all support above conclusions. Heterogeneity analysis demonstrates that males, older workers, ethnic minorities and those with lower health and socioeconomic status experience higher levels of depression. Highly educated and urban residents suffer from greater mental disorders after the turning point. Religious believers and Communist Party of China members are mentally healthier at lower income levels, meaning that religious and political beliefs moderate the relationship between income and mental health.

## Introduction

In the area of mental health, an almost universally accepted conclusion is that depression is inversely related to income (Thoits and Hannan, 1979). In earlier studies, the exact relationship between income and mental health can hardly been determined (Diener, 1984). Subsequent empirical tests confirm a statistically significant correlation between income and mental health (Diener et al., 1993). When depression is widely used to measure mental health, studies based on data from the United States and South Africa have further demonstrated that those with lower personal or household income tend to be at higher risk of depression (Sareen et al., 2011; Lund and Cois, 2018). Although studies find higher levels of depression in lower-income groups, after controlling other demographic, social and economic characteristics, the significant relationship between income and depression becomes less robust (Zimmerman and Katon, 2005). Similar results are obtained when measuring mental disorders in terms of pain and anger: low income tends to be significantly associated with the two kinds of negative emotions (Koltai et al., 2018). Studies have also found that the impact of income can be transmitted through subjective financial well-being (Iannello et al., 2021). This relationship is also more pronounced among low-income individuals and women (Chen et al., 2019; Zhang et al., 2020; Pan et al., 2021). Recent research finds that the relationship between income and subjective well-being is also related to the measurement of the two factors, which is why there are variations in the estimations on the relationship in existing research.

Existing literature also demonstrates that the correlation between income loss and depression persists over time. For example, some studies use personal past difficult experiences to study the lagging effect of financial hardship on mental health and find that those who experienced financial hardships have a higher risk of mental disorders than their counterparts (Kiely et al., 2015). In the COVID-19 epidemic, low-income workers face the risk of lower subjective well-being than their counterparts. The lockdown caused by the epidemic has also forced many people to furlough or even lose job. Those who were furloughed suffered more happiness loss than workers who were able to continue working (New York: UN Sustainable Development Solutions Network, 2021). This is also consistent with other research findings in the context of COVID-19, that income loss can negatively impact mental health (Tran et al., 2020; Hajek et al., 2022). In the latest *World Happiness Report*, data show that the COVID-19 outbreak significantly reduced both income and life satisfaction (New York: UN Sustainable Development Solutions Network, 2022). Income inequality is also an important factor. Empirical studies have found that in countries with higher income inequality, the relationship between income and mental well-being is more significant (Macchia et al., 2020). Furthermore, evidence from Japan shows that the relationship between income and subjective well-being rather than objective well-being is more prominent (Tsurumi et al., 2021).

Additionally, studies have shown that income has a diminishing marginal effect. This is because when the income reaches a certain threshold or the baseline, income would have already covered the basic necessities of life. According to Maslow’s hierarchy of needs theory, higher-level factors influence well-being. After this point, changes in happiness depend more on factors such as social relationship and self-actualization than on income (New York: UN Sustainable Development Solutions Network, 2012), which is why $10 has different effects on the poor and the rich. It is worth noting that it is more difficult for people who emphasize high income to raise happiness (Easterlin, 2001; Nickerson et al., 2003). Furthermore, based on data from the Gallup World Poll and the World Income database, the European sample demonstrates that lagged higher income is statistically significantly associated with lower average enjoyment and higher average stress and sadness (Powdthavee et al., 2017). This may explain the phenomenon of happiness not increasing in countries where incomes increased. Other research shows that decreasing income has a greater impact on mental health than rising income (Boyce et al., 2013; Shields-Zeeman and Smit, 2022). Recent studies demonstrate that income growth that lifts people out of poverty has a much higher positive impact on mental health than other types of income increase (Thomson et al., 2022). This implies that the relationship between income and mental health may not be simply linear.

Besides, studies also indicate the reverse causality between income and mental health. The 2012 *World Happiness Report* reveals that there is a bidirectional relationship between income and well-being (New York: UN Sustainable Development Solutions Network, 2012) and higher well-being is associated with higher current income (Judge et al., 2010) and future income (Marks and Fleming, 1999; Diener et al., 2002; Graham et al., 2004). Research based on a large representative dataset in the United States finds that those who report higher life satisfaction during their teenage years have significantly higher income levels as adults. This effect includes both direct and indirect impacts, mainly transmitted through college enrollment, opportunities of being hired and levels of optimism and extraversion (De Neve and Oswald, 2012). In addition to this, it is found that poor mental health may affect people’s ability to engage in paid work and thus income (Benzeval et al., 2014). Moreover, worse socioeconomic conditions lead to mental illnesses such as depression and social withdrawal, further impairing people’s human capital, work ability, and income. Mental problems such as depression and anxiety can directly affect the way people think by distracting attention, which in turn may further distort their economic preferences and decision-making (Ridley et al., 2020). In addition, lack of concentration and fatigue caused by depression can also reduce work efficiency, lower people’s performance in the labor market, and thus affect income. The loss of income due to depression may further impact future mental health, leading to a vicious circle (Elwell-Sutton et al., 2019). In this regard, provision of health or unemployment insurance can reduce depression and anxiety to a certain extent (Hynek et al., 2021). However, there are also findings suggesting that the causal relationship between income and mental health is uncertain. While lower income is associated with higher levels of depression, the extent to which income has a causal effect is debated (Thomson et al., 2021; Shields-Zeeman and Smit, 2022).

By reviewing relevant existing research above, it is not difficult to find that most of the existing literature assumes that there is a linear positive correlation between income and mental health (Lund et al., 2010; Brenner et al., 2014; Tampubolon and Hanandita, 2014; Barbaglia et al., 2015; Clingingsmith, 2016; Horn et al., 2017; Lachowska, 2017; Kilburn et al., 2018; Arvind et al., 2019; Swift et al., 2020). Moreover, on the one hand, since the existing literature shows that income has a diminishing marginal effect on mental well-being (New York: UN Sustainable Development Solutions Network, 2012), we are inspired to examine the nonlinear effect of income when exploring the relationship between the two factors. On the other hand, because studies have shown that there is a bidirectional causal relationship between income and well-being, endogeneity needs to be addressed to scientifically measure the effect of income on depression. In view of this, this research aims to examine the nonlinear effect of income on mental disorders based on tackling the endogeneity problem. Based on a large-scale nationally representative dataset, Chinese General Social Survey (CGSS), this paper uses the exogenous shock to income from automation as the instrument variable to explore the causal impact of income on mental health and explores the nonlinear influence of income on depression by including higher-order terms of income in regressions. A series of robustness checks are also conducted. Furthermore, we find that the nonlinear effect of income is heterogeneous in terms of gender, age, education, region, socioeconomic status, and social identity.

## Materials and methods

### Participants

Participants are from a large-scale nationally representative survey, Chinese General Social Survey (CGSS), conducted in 2017 and 2018. CGSS is one of the most important national, comprehensive and continuous academic survey projects in China. CGSS is in the world General Social Survey family, jointly carried out jointly by Renmin University of China and Hong Kong University of Science and Technology. The sampling of CGSS is based on the multi-stage stratified design. It aims to gather data on Chinese society to monitor and explain trends in income, attributes, behaviors, health and attitudes to examine the structure and functioning of society in general. Details of the study protocol and data files are introduced in the Supplementary material and available on http://cgss.ruc.edu.cn/English/Home.htm.

### Methods

Unraveling the effect of income on mental health is difficult because randomized controlled trials of income are almost not available. The main endogeneity issue here is reverse causality, whereby mental health also affects income (Ridley et al., 2020; Shields-Zeeman and Smit, 2022). Therefore, their relationship detected using observational data in the existing literature is more of a correlation than causality. This paper innovatively uses the exogenous impact of automation on income as the instrument variable to explore the causal effect of income on mental health based on dealing with endogeneity, especially the reverse causality. In addition, to examine the nonlinear relationship between income and depression, we use both income and its square as independent variables. Specifically, the following Two Stage Least Square (2SLS) econometric model is constructed.

$$\begin{array}{l} \ln\_\mathrm{Incom}e_{i}=\alpha_{0}+\alpha_{1}\mathrm{Automatio}n_{i}+\\ \;\alpha_{2}\mathrm{Automation}\_\mathrm{square}d_{i}+\overset{K}{\underset{k=1}{\sum }}\delta_{k}^{1}x_{ki}+\lambda_{t}+\theta_{p}+\varepsilon_{i} \end{array}$$

$$\begin{array}{l} \ln\_\mathrm{Income}\_\mathrm{square}d_{i}=\beta_{0}+\beta_{1}\mathrm{Automatio}n_{i}+\\ \;\beta_{2}\mathrm{Automation}\_\mathrm{square}d_{i}+\overset{K}{\underset{k=1}{\sum }}\delta_{k}^{2}x_{ki}+\lambda_{t}+\theta_{p}+\epsilon_{i} \end{array}$$

$$\begin{array}{l} \mathrm{Dpressio}n_{i}=\gamma_{0}+\gamma_{1}{\hat{\ln\_\mathrm{Income}}}_{i}+\\ \;\gamma_{2}\hat{\ln\_\mathrm{Income}\_\mathrm{square}d_{i}}+\overset{K}{\underset{k=1}{\sum }}\delta_{k}^{3}x_{ki}+\lambda_{t}+\theta_{p}+\mu_{i} \end{array}$$

In the model, ln_Income and ln_Income_squared are the logarithm of income and its square. Automation and Automation_squared are the indicators of the degree of automation’s influence on income and its quadratic term. Dpression represents the participant’s depression level. $x_{ki}$ is a series of control variables. $\lambda_{t}$ and $\theta_{p}$ control time and provincial fixed effects. We use the first two equations to conduct the first stage estimation of ln_Income and ln_Income_squared , and obtain $\hat{\ln\_\mathrm{Income}}$ and $\hat{\ln\_\mathrm{income}\_\mathrm{squared}}$ , respectively. In the second stage estimation, the third equation is regressed using $\hat{\ln\_\mathrm{Income}}$ and $\hat{\ln\_\mathrm{income}\_\mathrm{squared}}$ to examine the nonlinear effect of income on mental health.

In this 2SLS model, the instrument variable is Automation . This index is constructed by Autor and Dorn (2013) and serves as the most commonly used indicator to characterize automation’s impact on labor income (Acemoglu and Restrepo, 2020). It has been proved that the higher the extent of job replacement by automation, the lower the income (Hubmer and Restrepo, 2022). Therefore, this instrument variable satisfies the correlation requirement. At the same time, due to the following three reasons, this instrument variable meets the exogeneity prerequisite. First, the replacement of jobs by automation and thus its impact on income are caused by exogenous technological progress, independent of workers’ individual-level characteristics. Second, the automation degree of occupations is higher-level than the individual-level characteristics of workers. Therefore, automation is exogenous to the individual-level regression of depression. Third, Autor and Dorn (2013) measure the automation index utilizing the task characteristics of different occupations in the *Dictionary of Occupational Titles* by the United States Department of Labor in 1977. Because the individual traits of current workers could not affect the characteristics of occupations in 1977, this instrument variable well satisfies the exogeneity condition from the perspective of avoiding reverse causality. Therefore, this paper can use above 2SLS model to scientifically investigate how income affects mental health based on dealing with endogeneity.

This measure ranges from-6.190 to 4.235. According to this index, the Automation indicator of those in sales and service elementary occupations, laborers in mining, construction, manufacturing and transport, and personal and protective service workers are equal or close to 4.235. This means that people’s jobs in these occupations are replaced by automation to a larger extent, and consequently their income are more negatively affected by the exogenous AI technological progress. On the contrary, corporate managers, physical, mathematical and engineering professionals, life science and health professionals and other professionals’ Automation measure are equal or close to the minimum of-6.190. This means that those in above occupations are complementary to automation applications, and their income is positively affected by automation technology. A detailed description of this index is provided in the Supplementary material.

### Measures

The explained variable used in this paper is the depression level of the individual. This variable is based on the classic Likert scale from 1 to 5 to characterize people’s depression level. In CGSS, this indicator comes from respondents’ intensity of feeling depressed, from not depressed, mildly depressed, moderately depressed, very depressed to severely depressed. This indicator is also one of the most commonly used depression indicators in the existing literature (Derogatis, 1978; Foa et al., 1998; Mohr et al., 1999; Beck and Gable, 2000; Horigian et al., 2021). The explanatory variable of this paper is the logarithm of individual income and its quadratic term. Referring to the literature on the determinants of depression (e.g., Rosenquist et al., 2011; Rai et al., 2013; Ustun, 2021), we comprehensively control the basic demographic characteristics, human capital characteristics, social characteristics, working characteristics, family characteristics and regional and time fixed effects to minimize omitted variable bias in regressions. (1) Basic demographic characteristics include age, the square of age and gender. (2) Human capital characteristics include education level, health status and whether she/he is a migrant. (3) Social characteristics include whether the respondent’s Hukou is in urban,^1^ whether she/he belongs to ethnic minorities, whether she/he is a religious believer and Communist Party of China (CPC) member. (4) Working characteristics include weekly working hours, whether the respondent has pension and medical insurance. (5) Family characteristics include whether the respondent is married, her/his number of children, number of houses and the family’s overall socio-economic status. (6) Regional and time characteristics include provincial and time dummies. The descriptive statistics of above variables are shown in Table 1.

**Table 1:** Descriptive statistics.

Variable	Description	Obs	Mean	Std. Dev.	Min	Max
**Dependent variable**						
Depression	1–5 levels	11,894	2.151	0.954	1	5
**Explanatory variables**						
ln_Income	Logarithm of household income (RMB)	11,913	9.450	2.774	0	16.113
ln_Income_squared	Square of logarithm of household income (RMB)	11,913	96.993	35.722	0	259.632
**Instrument variables**						
Automation	Automation index	11,913	−0.461	1.333	−6.190	4.235
Automation_squared	Square of the automation index	11,913	1.987	4.151	0	38.314
**Demographic characteristics**						
Age	Age	11,913	45.452	13.135	18	75
Age_squared	Squared term of age	11,913	2238.403	1219.181	324	5,625
Whether female	Yes = 1, No = 0	11,913	0.468	0.499	0	1
**Human capital characteristics**						
Education level	1–13 levels	11,909	5.483	3.389	1	13
Health status	1–5 levels	11,909	3.688	1.026	1	5
Whether migrants	Yes = 1, No = 0	11,885	0.155	0.362	0	1
**Social characteristics**						
Whether Hukou in urban	Yes = 1, No = 0	11,890	0.300	0.458	0	1
Whether ethnic minorities	Yes = 1, No = 0	11,913	0.081	0.272	0	1
Whether religious believer	Yes = 1, No = 0	11,913	0.095	0.293	0	1
Whether CPC member	Yes = 1, No = 0	11,902	0.102	0.3024	0	1
**Working characteristics**						
Weekly working hours	Working hours per week	11,913	43.915	23.376	0	88
Whether having pension	Yes = 1, No = 0	11,898	0.742	0.437	0	1
Whether having medical insurance	Yes = 1, No = 0	11,908	0.935	0.247	0	1
**Family characteristics**						
Whether married	Yes = 1, No = 0	11,913	0.806	0.395	0	1
Family size	Number of members in the family	11,904	2.947	1.518	1	44
Number of children	Number of children in the family	11,902	1.523	1.094	0	20
Number of houses	Number of houses in the family	11,845	1.119	0.661	0	11
Socio-economic status	Whether in the middle-upper class of the society	11,834	0.497	0.500	0	1
Year dummy						
Province dummies						

The education level is classified from 1 to 13: 1-without any education, 2-kindergarten, 3-primary school, 4-junior high school, 5-vocational high school, 6-ordinary high school, 7-techinical secondary school, 8-technical high school, 9-junior college (adult higher education), 10-junior college (regular higher education), 11-undergraduate (adult higher education), 12-undergraduate (regular higher education), 13-postgraduate and above. Health status is based on the classic Likert scale from 1 to 5 to represent 1-unhealthy, 2-relatively unhealthy, 3-general, 4-relatively healthy, 5-healthy. The meanings of dummy variables are all “Yes = 1, No = 0.”

## Results

### Benchmark results

Estimation results using above 2SLS model are demonstrated in Table 2. Column (1) is the regression without any control variables, showing that both of ln_Income and ln_Income_squaered ’s estimated coefficients are statistically significant. The estimate of ln_Income is significantly negative, whereas that of ln_Income_squaered is positive. This means that income’s effect on depression is nonlinear. Columns (2)–(6) are regression results gradually adding demographic characteristics, human capital characteristics, social characteristics, working characteristics, family background and regional and time dummies. It is shown that in all regressions, estimates of income and its squared term are all significant, indicating that the nonlinear effect of income on depression is very robust. In addition, with the addition of different types of control variables, estimates of the two variables are basically stable at around-1.4 and 0.1, respectively. This means that the nonlinear relationship between income and mental health is almost not affected by other factors.

**Table 2:** U-shaped effects of income on depression.

Model	(1) 2SLS	(2) 2SLS	(3) 2SLS	(4) 2SLS	(5) 2SLS	(6) 2SLS	(7) 2SLS
Variable	Depression	Depression	Depression	Depression	Depression	Depression	Depression
ln_Income	−1.064^***^ (0.394)	−0.837^**^ (0.361)	−1.506^**^ (0.601)	−1.499^**^ (0.595)	−1.635^**^ (0.715)	−1.401^**^ (0.636)	1.585 (2.906)
ln_Income_squared	0.062^**^ (0.024)	0.050^**^ (0.023)	0.100^**^ (0.039)	0.100^***^ (0.039)	0.103^**^ (0.044)	0.091^**^ (0.039)	−0.323 (0.394)
ln_Income_cubed							0.016 (0.015)
Age		0.023^*^ (0.012)	−0.000 (0.016)	0.002 (0.016)	0.013 (0.021)	0.016 (0.017)	0.014 (0.014)
Age_squared		−0.000 (0.000)	0.000 (0.000)	0.000 (0.000)	−0.000 (0.000)	−0.000 (0.000)	−0.000^*^ (0.000)
Whether female		0.013 (0.066)	−0.052 (0.087)	−0.048 (0.089)	−0.095 (0.117)	−0.013 (0.101)	0.095 (0.161)
Education level			−0.081^***^ (0.024)	−0.064^***^ (0.022)	−0.043^**^ (0.019)	−0.026^*^ (0.014)	−0.017^*^ (0.009)
Health status			−0.315^***^ (0.026)	−0.314^***^ (0.027)	−0.291^***^ (0.036)	−0.283^***^ (0.029)	−0.274^***^ (0.020)
Whether migrants			−0.208^**^ (0.089)	−0.221^**^ (0.090)	−0.192*^*^ (0.092)	−0.044 (0.059)	0.032 (0.069)
Whether Hukou in urban				−0.149^***^ (0.055)	−0.076 (0.069)	−0.033 (0.051)	0.003 (0.037)
Whether ethnic minorities				0.159^*^ (0.084)	0.154^*^ (0.092)	−0.095 (0.061)	−0.143^***^ (0.055)
Whether religious believer				0.089 (0.058)	0.121^*^ (0.072)	0.167^**^ (0.078)	0.061 (0.122)
Whether CPC member				−0.156^***^ (0.047)	−0.137^***^ (0.049)	−0.119^***^ (0.044)	−0.068 (0.051)
Weekly working hours					0.008^**^ (0.004)	0.007^*^ (0.003)	0.002 (0.006)
Whether having pension					−0.005 (0.051)	0.034 (0.056)	−0.047 (0.095)
Whether having medical insurance					0.049 (0.079)	0.063 (0.071)	0.036 (0.053)
Whether married						−0.113^**^ (0.055)	−0.126^***^ (0.047)
Family size						−0.016 (0.014)	0.010 (0.028)
Number of children						−0.018 (0.023)	−0.020 (0.015)
Number of houses						−0.066^*^ (0.037)	−0.053^**^ (0.026)
Socio-economic status						−0.210^***^ (0.039)	−0.170^***^ (0.034)
Year dummy	No	No	No	No	No	Yes	Yes
Province dummies	No	No	No	No	No	Yes	Yes
Constant	6.201^***^ (1.374)	4.401^***^ (1.021)	8.259^***^ (1.766)	8.030^***^ (1.726)	8.210^***^ (1.999)	6.783^***^ (1.794)	2.975 (3.995)
Observations	11,894	11,894	11,858	11,830	11,816	11,655	11,655

^***^, ^**^, and ^*^ indicate significance at the levels of 1%, 5%, and 10%, respectively. The values in parentheses are standard errors robust to heteroskedasticity. Yes means the corresponding variables are controlled in the regression, while No means not controlled.

**Figure. 1:**
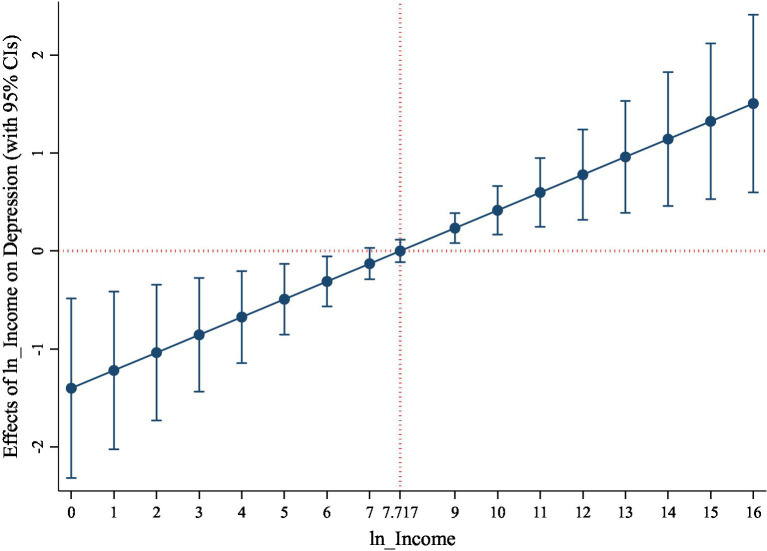
Marginal effects of income on depression.

Specifically, according to the results in column (6) of Table 2, the estimates of income and its square are-1.401 and 0.091, indicating a U-shaped effect of income on depression. This means that in the lower-income level, depression declines as income increases. This is mainly due to the fact that the higher the income, the higher the living standards, which are conducive to improving mental health. However, after income increases to a certain level, amounting to 7.717 (=1.401499/(0.0908046*2)), depression rises with the increase of income. In CGSS, the range of ln_Income is [0, 16.113]. Therefore, after the middle income, further increases in income raise depression levels. The underlying reason is that the negative effects of working efforts required to further improve income are greater than the benefits of rising earnings, causing the increase of mental disorders. This confirms that the relationship between income and mental health is a nonlinear U-shaped relationship. Figure 1 intuitively shows that as income increases, its effect on depression is firstly negative and then positive.

Based on the above analysis, we naturally further propose the question: Whether the relationship between income and depression is a U-shaped relationship or a more complex one with more turning points, such as a W-shaped or M-shaped relation. We test this hypothesis by further incorporating the cubic term of income, ln_Income_cubed , into the model to conduct a similar 2SLS estimation. Regression results shown in column (7) of Table 2 demonstrate that after this cubic term is included, all the estimated coefficients of the three variables of income are not significant. This means that there are strong multicollinearities among the three variables, causing the standard error of the estimation too large. Therefore, ln_Income_cubed should not be included into the regression. This also confirms that the impact of income on depression is not cubic or other nonlinear relations with more turning points.

### Robustness checks

We conduct robustness checks on the U-shaped effects of income on mental health in the following four aspects. First, the sample sizes are different from Columns (1) to (6). The reason is that the numbers of different control variables’ observations are slightly different. Consequently, as more control variables are added in the regressions, sample size decreases to some extent. Naturally, we are concerned whether this affects the analytical results of this paper. We further conduct a robustness test and the results are shown in Supplementary Table 1. It is obvious that when we use observations with no missing values in all control variables and the sample size keeps constant in all regressions, estimation results are almost consistent with above benchmark results. This means that the variation of sample sizes caused by missing values of controls does not affect the conclusions. Second, we perform regressions using another measure of automation as the instrument variable. This index comes from Marcolin et al. (2019) and is calculated according to the frequencies with which individuals in a certain occupation may use automation to finish the work. The higher the indicator, the greater the impact of automation on workers’ income. Regression results using this IV are shown in Supplementary Table 2, which are consistent with the benchmark analysis. This means that the U-shaped relationship between income and mental health is robust and does not rely on the selection of the automation IV. Third, we use “whether feel depression” as the explained variable to investigate the impact of income on it. Supplementary Table 3 demonstrates that both income and its square have significant effects on “whether feel depression,” further confirming the U-shaped relationship between income and depression. Fourth, we conduct regressions using other types of IV models, including limited information maximum likelihood estimation (LIML), efficient two-step generalized method of moments (GMM) and iterative GMM (IGMM). Results in Supplementary Table 4 demonstrate that regardless of the instrument variable regression methods used, the nonlinear effect of income on depression is robustly supported. Fifth, we carry out a placebo test of the analytical results. Specifically, we randomly reassign ln_Income and ln_Income_squared in the sample. The randomization ensures that in the new sample the two variables should have no significant effect on depression and the *p* values of their estimates should be larger than 0.1. Supplementary Figure 1 illustrates the p values of the ln_Income and ln_Income_squared ’s estimated coefficients in 1000 such placebo samples, in which most of the p values are larger than 0.1. This confirms that our results are not caused by random omitted factors and the U-shaped relationship between income and depression is robust.

### Heterogeneity analysis

**Figure 2:**
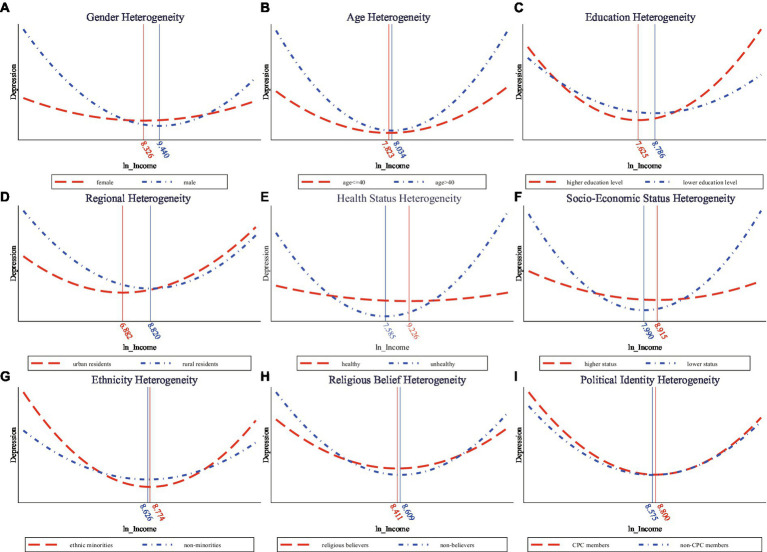
**(A-I)** Heterogeneities in the effects of income on depression.

This paper further investigates the heterogeneity of the U-shaped relationship between income and depression in multiple aspects and the results are illustrated in Figure 2. In terms of gender, it is shown that men are generally more depressed than women. Especially in the lower income stage, men bear significantly more mental health burden. Furthermore, the turning point of the U-shaped relationship is greater for men (9.440) than for women (8.326). This means that men are more willing to take on pressure to increase their income. This feature is related to the Chinese tradition of “Men take charge and women take care.” In terms of age heterogeneity, the findings suggest that older workers have higher levels of depression. In addition, the U-shaped curve between income and depression is steeper in the older age group. This means that for older workers, the cost of mental disorders in raising income beyond the turning point is much higher. In respect of education, it is illustrated that those with a bachelor’s degree or above are mentally healthier at the lower-income interval. However, after the turning point, they tend to suffer from higher levels of depression.

With regard to regional heterogeneity, results show that below the turning point, urban residents have lower levels of depression. But at the high-income stage, they take on more burden of mental disorders. This may be caused by the fact that the urban labor market is more competitive and the mental health costs of improving incomes are higher. As to health heterogeneity, self-rated healthy respondents have lower levels of depression and a much bigger turning point of the U-shaped relationship. This means that the overall health status is closely associated with mental health and those with higher overall health status tend to be mentally healthier. Regarding socioeconomic status, the higher socioeconomic status group is less depressed. Its turning point of the nonlinear relationship is much larger and the U-shaped curve is relatively flatter. This means lower mental health costs to improve income for the higher socioeconomic group.

Concerning social identity, we examine the heterogeneities in the aspects of ethnicity, religious belief and political identity. Analytical results show that ethnic minorities have higher levels of depression. Especially in the high-income stage, their mental health cost to further increase income is obviously higher. In general, religious believers’ mental health condition is better. Moreover, there is no significant difference in depression between Chinese Communist Party (CPC) members and non-CPC members. However, in the lower income interval, CCP members have higher levels of mental health. This may be due to the fact that CCP members are educated and required to sacrifice selflessly, making them easier to accept lower income.

## Conclusion and discussion

Using a large-scale nationally representative survey, the Chinese General Social Survey (CGSS), this paper systematically explores the relationship between income and mental health. In terms of research methods, we use the exogenous impact of automation on income as the instrument variable and investigate income’s impact on depression based on scientifically dealing with endogeneity. In addition, to examine the nonlinear relationship between income and depression, we analyze both income and its quadratic term in regressions. Results demonstrate that the estimated coefficients of income and its square are statistically negative and positive, respectively. This means that the relationship between income and mental health is U-shaped rather than a simple linear correlation. The turning point (7.698) of the U-shaped effect is close to the midpoint of the income interval ([0, 16.113]). This suggests that, in lower levels of income, depression declines as income rises. However, beyond the turning point around middle income, further increases in income bring about notable mental health costs, leading to a positive relationship between the two factors. Findings of the nonlinear causal relationship contribute to the existing literature where a simple linear correlation between income and mental health is assumed. In addition, we also exclude the possibility of more complex nonlinear relationships between income and mental health. Moreover, robustness tests are carried out, such as using other types of instrument variables, mental health indicators and IV models, as well as placebo analysis. Their results further confirm that the U-shaped effects of income on mental health are robust.

Most of existing studies conclude that there is a linear relationship between income and mental health, which is proved to be positive in both earlier studies (e.g., Diener et al., 1993; Marks and Fleming, 1999; Graham et al., 2004; Judge et al., 2010; Lund et al., 2010; Sareen et al., 2011; Sun et al., 2011; De Neve and Oswald, 2012) and more recent ones (e.g., Arvind et al., 2019; Elwell-Sutton et al., 2019; Macchia et al., 2020; Ridley et al., 2020; Swift et al., 2020; Tran et al., 2020; Hertz-Palmor et al., 2021; Hynek et al., 2021; New York: UN Sustainable Development Solutions Network, 2021; Pan et al., 2021; Su et al., 2021; Thomson et al., 2021; Hajek et al., 2022; Shields-Zeeman and Smit, 2022; Thomson et al., 2022). The conclusion drawn in this paper that income has a U-shaped impact on depression is consistent with findings of these studies to some extent, because as income increases mental health improves within a certain range of income. However, we further find that this positive relationship holds only below a specific income level. When income rises to a threshold, further increase in income takes significant mental health costs. From this perspective, this paper revises and extends above literature that has only considered a linear relationship between income and mental health. Moreover, this research rejects a few papers’ conclusions that there is no significant relationship between income and mental health (e.g., Zimmerman and Katon, 2005). The reason for the insignificance may be due to the fact that these studies do not consider the quadratic term of income in their empirical tests. Therefore, the positive impact of income on mental health at the lower-income stage and the negative impact after the turning point are offset against each other, leading to a seemingly insignificant relationship.

Furthermore, after carefully and thoroughly searching the literature in many ways, three existing studies are found which indirectly imply a nonlinear relationship between income and mental disorders. One of them comes from Chapter 3 of New York: UN Sustainable Development Solutions Network (2012)*: The Causes of Happiness and Misery*. The research in this report analyzes the possible nonlinear relationship between income and mental health from a theoretical perspective, that is, income positively affects mental health with diminishing marginal returns (New York: UN Sustainable Development Solutions Network, 2012). However, it does not conduct empirical tests. Another study examines the effect of the spouse’s income on individual mental health (Syrda, 2020). Specifically, it uses the husband’s psychological distress as the dependent variable and wife’s relative income as the independent variable. The results demonstrate that men’s distress firstly decreases with their wives’ income rising. However, after the wife’s income reaches 40% or above of the household total income, male psychological distress increases. This is because the emotional burden of the husband’s declining relative status in the household outweighs the benefits of higher total income for men. Nevertheless, this study does not explore the nonlinear effects of people’s own income on her or his mental health. Besides, another paper conducts an empirical analysis using current income as the dependent variable and cheerfulness as the independent variable (Diener et al., 2002). It is found that income firstly rises as cheerfulness increases. However, after cheerfulness reaches a certain threshold, income no longer improves with cheerfulness, but tends to level off and eventually even decreases slightly. This study, while implying a nonlinear relationship between mental status and income, is not an exploration of the effect of income on mental health. In summary, very few studies have considered the possible nonlinear association between income and mental health to some extent. However, there is no literature directly empirically examining the U-shaped impacts of income on mental disorders at the individual level. Our study proposes the hypothesis that the relationship between income and depression may be nonlinear based on theoretical analysis and conducts a systematic empirical investigation. We find that when income is low, increasing income helps reduce mental disorders, but after income rises to a certain level, further growth in income brings about considerable mental health costs, leading to a nonlinear relationship between the two factors. This is the main contribution of this paper.

Besides, this paper conducts a detailed analysis on the heterogeneity of the U-shaped relationship between income and depression in multiple aspects. It is shown that men, older workers, minority groups and those with lower health and socioeconomic status experience higher levels of depression at nearly all income levels compared with their counterparts. These findings are consistent with existing literature. For example, it is concluded that groups with lower socioeconomic status have higher rates of mental illness (Lorant et al., 2007; Reiss, 2013). In addition, during the great recession, individuals with lower levels of health status, such as illness or disability, experience much greater decline in well-being (Boyce et al., 2018). It is also found that ethnic minorities tend to be disadvantaged in terms of mental health and subjective well-being even earning the same income with other groups (Diener et al., 1993). In terms of gender heterogeneity, Kilburn et al. (2016) demonstrate that the effect of increasing income on depression is more pronounced for the younger male. In addition, our study shows that mental health costs of further raising income after the turning point are higher for those with higher education levels and urban workers. Existing studies have found that groups with lower income levels are more susceptible to psychiatric problems (Cheung and Lucas, 2015; Cooper and Stewart, 2015; Ridley et al., 2020; Thomson et al., 2022), while the role of religious and political identity is not fully emphasized. In comparison, our study demonstrates that religious believers have higher levels of mental health. Besides, although generally no significant difference in mental health between CPC members and non-CPC members is detected, CPC members are less depressed at the lower income level. These findings imply that religious and political beliefs play a part in moderating the relationship between income and mental health.

We further compare the results of this paper with existing studies based on Chinese samples as well as those in other countries from the perspective of international comparisons. The literature reveals that studies focusing on China in this area are very limited. It also means that this study contributes to the literature by providing novel evidence on the relationship between income and mental disorders among Chinese people. Existing studies using Chinese samples (e.g., Sun et al., 2011; Bian et al., 2015) mainly utilize mental health measured by anxiety and depression or subjective well-being as the dependent variables. Their results all show that there is a significantly positive relationship between income and mental health. In addition, some studies analyze the impact of pension, the major source of income for the elderly, on older people’s mental health (Chen et al., 2019; Pan et al., 2021). It is also concluded that there is a linear positive relationship between pension enrollment and mental health for older persons. The main reason for why this research finds a nonlinear relationship between income and mental health is that we further examine the effect of the quadratic term of income on depression.

In addition, the particularity of the relationship between income and depression in China should be emphasized, which may be reflected in two aspects. First of all, China is a middle-income developing country. Therefore, compared with those in developed economies, Chinese people may have a more urgent need to further improve their income, a greater tolerance for stress caused by work, and thus a higher turning point in the relationship between income and depression. But compared to countries with lower *per capita* income, the turning point for Chinese people may be lower. Therefore, for other countries, whether there exists a nonlinear relationship between income and depression, and whether the turning point varies with different levels of economic development, is a valuable direction for further research. Second, China is a country profoundly influenced by Confucianism, a culture that emphasizes that people should bear working pressure, contribute to their family and be responsible for both supporting their parents and raising children. Therefore, Chinese people may have a higher tolerance for working pressure and working hours, which may also lead to a larger turning point in the Chinese sample. In view of this, an international comparative study of the nonlinear relationship between income and depression based on the comparable microdata of China and other countries would be a very meaningful research direction.

Clinical implications of the study include: First, our results reveal that the relationship between income and mental health is U-shaped rather than a simple linear correlation. This confirms that when people’s income exceeds the median income, further increase in income takes considerable mental health costs. From clinical experience, it is also found that the depression problem in the high-income group is very prominent because of increased work pressure. Therefore, for people with higher income, it is of great importance to be aware of the mental health costs of further raising income. It is crucial to rationally weigh the costs and benefits between income and mental health in the high-income stage. Second, according to the heterogeneity results of this paper, more attention should be paid to the vulnerable subgroups in terms of mental health in clinical practice. For example, men and older workers are generally more depressed than women, meaning that they take on more work pressures and their mental health problems should receive more attention. In addition, depression problems before the turning point are mainly reflected in individuals with lower educational levels, but the mental health risks of people with higher education in the high-income stage should be emphasized. Furthermore, mental health problems in those with lower overall health levels and socioeconomic status and ethnic minorities are more prominent. Therefore, their depressive disorders need to be focused on in clinical practice.

This paper has the following shortcomings. First, this study uses a cross-sectional data, rather than panel data. Although the instrument variable method is used to solve the endogeneity problem, the panel data can further control individual fixed effects and so the omitted variable bias can be better solved. The reason why this study does not use longitudinal data is that the existing panel datasets does not include the necessary explained or instrument variables used in this paper. Therefore, we look forward to a further study of the relationship between income and mental health based on better panel data in the future. Second, this paper proves the nonlinear relationship between income and mental health but fails to further examine the underlying mechanisms of the U-shaped relationship. We have proposed several possible mechanisms, which could hardly be tested due to the lack of relevant variables in the data. Third, scales like PHQ-9 are better indicators to measure the degree of depression and contain more comprehensive information than self-rated depression levels. However, this is not available in the CGSS dataset, which is also a shortcoming of this study. It would be meaningful to collect specific data for this topic to further explore the underlying mechanisms of the nonlinear relationship between income and mental health.

## Data availability statement

The original contributions presented in the study are included in the article/Supplementary material, further inquiries can be directed to the corresponding author.

## Ethics statement

The studies involving human participants were reviewed and approved by the Institutional Review Board, Renmin University of China. The patients/participants provided their written informed consent to participate in this study.

## Author contributions

CL contributed to the conception and design of the study, performed the statistical analysis, and wrote the first draft of the manuscript. GN, LW, and FC generated the tables and figures based on CL’s analysis. GN, LW, and FC worked on revisions of the manuscript. All authors contributed to the article and approved the submitted version.

## Funding

This work was supported by the Humanities and Social Science Research Project of the Ministry of Education of China (grant number 19YJC790055), the Project of Natural Science Foundation of China (grant number 71973081), the Project of Natural Science Foundation of Shandong Province, China (grant number ZR2020QG038), the Project of Social Science Foundation of Shandong Province, China (grant number 19DJJJ08), and the Project of Teaching Reform of Shandong University (grant number Y2022007).

## Conflict of interest

The authors declare that the research was conducted in the absence of any commercial or financial relationships that could be construed as a potential conflict of interest.

## Publisher’s note

All claims expressed in this article are solely those of the authors and do not necessarily represent those of their affiliated organizations, or those of the publisher, the editors and the reviewers. Any product that may be evaluated in this article, or claim that may be made by its manufacturer, is not guaranteed or endorsed by the publisher.

## Footnotes

^1^Hukou is a system of household registration used in mainland China, mainly identifying a person as a rural or urban resident.
