# Supplementary material for: More income, less depression? Revisiting the nonlinear and heterogeneous relationship between income and mental health
Source: Front Psychol. 2022 Dec 14;13:1016286. doi: 10.3389/fpsyg.2022.1016286 (PMC9795204; doi:10.3389/fpsyg.2022.1016286)
Supplement: Supplementary file 1 [file Data_Sheet_1.pdf]

Supplementary Material

Supplementary Table 1. Robustness Checks Using Consistent Samples

Model<br>Variable	(1) 2SLS<br>Depression	(2) 2SLS<br>Depression	(3) 2SLS<br>Depression	(4) 2SLS<br>Depression	(5) 2SLS<br>Depression	(6) 2SLS<br>Depression	(7) 2SLS<br>Depression
ln_Income	-1.196***	-0.945**	-1.655**	-1.621**	-1.774**	-1.401**	1.585
	(0.436)	(0.396)	(0.660)	(0.644)	(0.787)	(0.636)	(2.906)
ln_Income_ squared	0.070***	0.058**	0.110**	0.109***	0.112**	0.091**	-0.323
	(0.027)	(0.025)	(0.043)	(0.042)	(0.048)	(0.039)	(0.394)
ln_Income_cubed							0.016
							(0.015)
Age		0.024*	-0.001	0.002	0.014	0.016	0.014
		(0.013)	(0.017)	(0.017)	(0.022)	(0.017)	(0.014)
Age_squared		-0.000	0.000	0.000	-0.000	-0.000	-0.000*
		(0.000)	(0.000)	(0.000)	(0.000)	(0.000)	(0.000)
Whether female		0.008	-0.060	-0.056	-0.105	-0.013	0.095
		(0.071)	(0.094)	(0.096)	(0.127)	(0.101)	(0.161)
Education level			-0.086***	-0.069***	-0.046**	-0.026*	-0.017*
			(0.026)	(0.023)	(0.020)	(0.014)	(0.009)
Health status			-0.316***	-0.314***	-0.292***	-0.283***	-0.274***
			(0.028)	(0.028)	(0.038)	(0.029)	(0.020)
Whether migrants			-0.231**	-0.250**	-0.220**	-0.044	0.032
			(0.099)	(0.100)	(0.103)	(0.059)	(0.069)
Whether Hukou in urban				-0.151***	-0.074	-0.033	0.003
				(0.058)	(0.074)	(0.051)	(0.037)
Whether ethnic minorities				0.157*	0.151	-0.095	-0.143***
				(0.088)	(0.096)	(0.061)	(0.055)
Whether religious believer				0.098	0.134*	0.167**	0.061
				(0.063)	(0.080)	(0.078)	(0.122)
Whether CPC member				-0.158***	-0.138***	-0.119***	-0.068
				(0.049)	(0.052)	(0.044)	(0.051)
Weekly working hours					0.009**	0.007*	0.002
					(0.004)	(0.003)	(0.006)
Whether having pension					0.000	0.034	-0.047
					(0.055)	(0.056)	(0.095)
Whether having medical insurance					0.062	0.063	0.036
					(0.086)	(0.071)	(0.053)
Whether married						-0.113**	-0.126***
						(0.055)	(0.047)
Family size						-0.016	0.010
						(0.014)	(0.028)
Number of children						-0.018	-0.020
						(0.023)	(0.015)
Number of houses						-0.066*	-0.053**
						(0.037)	(0.026)
Socio-economic status						-0.210***	-0.170***
						(0.039)	(0.034)
Year dummy	No	No	No	No	No	Yes	Yes
Province dummies	No	No	No	No	No	Yes	Yes
Constant	6.649***	4.682***	8.737***	8.418***	8.621***	6.783***	2.975
	(1.524)	(1.130)	(1.962)	(1.891)	(2.217)	(1.794)	(3.995)
Observations	11655	11655	11655	11655	11655	11655	11655

Supplementary Table 2. 2SLS Regression Results Using Marcolin et al. (2019)'s Automation Index as the Instrument Variable

Model<br>Variable	(1) 2SLS<br>Depression	(2) 2SLS<br>Depression	(3) 2SLS<br>Depression	(4) 2SLS<br>Depression	(5) 2SLS<br>Depression	(6) 2SLS<br>Depression	(7) 2SLS<br>Depression
ln_Income	-0.437***	-0.509***	-0.475***	-0.500***	-0.685***	-0.737***	0.053
	(0.152)	(0.177)	(0.168)	(0.168)	(0.227)	(0.248)	(3.980)
ln_Income_ squared	0.018**	0.022**	0.024**	0.026**	0.034***	0.041***	-0.068
	(0.009)	(0.010)	(0.010)	(0.010)	(0.013)	(0.014)	(0.547)
ln_Income_cubed							0.004
							(0.022)
Age		0.031***	0.015	0.017	0.022*	0.023	0.021
		(0.010)	(0.011)	(0.011)	(0.013)	(0.015)	(0.020)
Age_squared		-0.000***	-0.000*	-0.000*	-0.000*	-0.000	-0.000*
		(0.000)	(0.000)	(0.000)	(0.000)	(0.000)	(0.000)
Whether female		-0.050	-0.045	-0.050	-0.078	-0.022	0.015
		(0.051)	(0.046)	(0.051)	(0.064)	(0.082)	(0.221)
Education level			-0.012	-0.007	0.000	0.001	0.001
			(0.010)	(0.010)	(0.011)	(0.012)	(0.011)
Health status			-0.288***	-0.287***	-0.275***	-0.270***	-0.271***
			(0.017)	(0.018)	(0.023)	(0.024)	(0.023)
Whether migrants			0.012	0.006	0.005	0.034	0.047
			(0.039)	(0.044)	(0.048)	(0.041)	(0.070)
Whether Hukou in urban				-0.020	0.018	0.024	0.024
				(0.040)	(0.051)	(0.051)	(0.045)
Whether ethnic minorities				0.000	0.011	-0.120**	-0.131**
				(0.041)	(0.046)	(0.047)	(0.065)
Whether religious believer				0.092**	0.113**	0.128***	0.100
				(0.037)	(0.044)	(0.048)	(0.152)
Whether CPC member				-0.120***	-0.115***	-0.111***	-0.098
				(0.032)	(0.035)	(0.036)	(0.068)
Weekly working hours					0.006***	0.005**	0.004
					(0.002)	(0.002)	(0.007)
Whether having pension					0.000	0.018	-0.004
					(0.032)	(0.038)	(0.123)
Whether having medical insurance					0.058	0.068	0.062
					(0.050)	(0.051)	(0.054)
Whether married						-0.089*	-0.096
						(0.045)	(0.060)
Family size						-0.005	-0.005
						(0.034)	(0.034)
Number of children						-0.007	-0.007
						(0.015)	(0.013)
Number of houses						-0.016	-0.016
						(0.021)	(0.019)
Socio-economic status						-0.159***	-0.153***
						(0.033)	(0.035)
Year dummy	No	No	No	No	No	Yes	Yes
Province dummies	No	No	No	No	No	Yes	Yes
Constant	4.563***	4.116***	5.238***	5.230***	5.601***	5.196***	4.177
	(0.605)	(0.603)	(0.506)	(0.511)	(0.659)	(0.785)	(5.270)
Observations	11894	11894	11858	11830	11816	11655	11655

Supplementary Table 3. IV-Probit Regression Results Using Whe_depression as the Dependent Variable

Model<br>Variable	(1)<br>IV-Probit<br>Whe_<br>depression	(2)<br>IV-Probit<br>Whe_<br>depression	(3)<br>IV-Probit<br>Whe_<br>depression	(4)<br>IV-Probit<br>Whe_<br>depression	(5)<br>IV-Probit<br>Whe_<br>depression	(6)<br>IV-Probit<br>Whe_<br>depression
ln_Income	-0.800***	-0.705***	-1.000***	-1.005***	-0.232***	-0.989***
	(0.131)	(0.200)	(0.112)	(0.113)	(0.072)	(0.124)
ln_Income_ squared	0.047***	0.044***	0.068***	0.069***	0.013**	0.066***
	(0.009)	(0.013)	(0.008)	(0.008)	(0.006)	(0.008)
Age		0.017*	-0.004	-0.003	-0.003	0.007
		(0.009)	(0.012)	(0.012)	(0.016)	(0.011)
Age_squared		-0.000	0.000	0.000	-0.000	-0.000
		(0.000)	(0.000)	(0.000)	(0.000)	(0.000)
Whether female		0.051	-0.009	-0.006	0.087	0.021
		(0.072)	(0.059)	(0.062)	(0.068)	(0.082)
Education level			-0.057***	-0.047***	-0.013	-0.026**
			(0.013)	(0.011)	(0.017)	(0.011)
Health status			-0.224***	-0.226***	-0.413***	-0.216**
			(0.083)	(0.084)	(0.037)	(0.098)
Whether migrants			-0.107**	-0.116**	0.062	-0.018
			(0.044)	(0.047)	(0.066)	(0.042)
Whether Hukou in urban				-0.099**	-0.069	-0.030
				(0.049)	(0.067)	(0.044)
Whether ethnic minorities				0.122***	0.012	-0.045
				(0.044)	(0.053)	(0.051)
Whether religious believer				0.079**	0.113**	0.126***
				(0.037)	(0.047)	(0.038)
Whether CPC member				-0.093**	-0.106**	-0.073*
				(0.039)	(0.048)	(0.042)
Weekly working hours					0.002*	0.004***
					(0.001)	(0.001)
Whether having pension					-0.024	0.021
					(0.036)	(0.036)
Whether having medical insurance					0.032	0.032
					(0.055)	(0.050)
Whether married						-0.097
						(0.067)
Family size						-0.007
						(0.009)
Number of children						-0.008
						(0.015)
Number of houses						-0.037*
						(0.021)
Socio-economic status						-0.171***
						(0.062)
Year dummy	No	No	No	No	No	Yes
Province dummies	No	No	No	No	No	Yes
Constant	2.696***	1.457**	3.815***	3.709***	2.099	3.048***
	(0.474)	(0.678)	(0.184)	(0.181)	(.)	(0.277)
Observations	11894	11894	11858	11830	11816	11655

Supplementary Table 4. Regression Results Using Different Instrument Variable Methods

Model	(1) First Stage	(2) First Stage	(3) 2SLS Second Stage	(4) LIML Second Stage	(5) GMM Second Stage	(6) IGMM Second Stage
Variable	ln_Income	ln_Income _squared	Depression	Depression	Depression	Depression
ln_Income			0.091**	0.091**	0.091**	0.091**
			(0.039)	(0.039)	(0.039)	(0.039)
ln_Income_ squared			-1.401**	-1.401**	-1.401**	-1.401**
			(0.636)	(0.636)	(0.636)	(0.636)
RII	-0.022	-0.634***				
	(0.018)	(0.215)				
RII_squared	0.035***	0.500***				
	(0.006)	(0.080)				
Age	0.139***	2.010***	0.016	0.016	0.016	0.016
	(0.014)	(0.161)	(0.017)	(0.017)	(0.017)	(0.017)
Age_squared	-0.002***	-0.024***	-0.000	-0.000	-0.000	-0.000
	(0.000)	(0.002)	(0.000)	(0.000)	(0.000)	(0.000)
Whether female	-0.837***	-11.594***	-0.013	-0.013	-0.013	-0.013
	(0.045)	(0.515)	(0.101)	(0.101)	(0.101)	(0.101)
Education level	0.133***	2.329***	-0.026*	-0.026*	-0.026*	-0.026*
	(0.009)	(0.113)	(0.014)	(0.014)	(0.014)	(0.014)
Health status	0.220***	3.089***	-0.283***	-0.283***	-0.283***	-0.283***
	(0.025)	(0.280)	(0.029)	(0.029)	(0.029)	(0.029)
Whether migrants	0.245***	4.857***	-0.044	-0.044	-0.044	-0.044
	(0.067)	(0.824)	(0.059)	(0.059)	(0.059)	(0.059)
Whether Hukou in urban	0.460***	7.132***	-0.033	-0.033	-0.033	-0.033
	(0.054)	(0.672)	(0.051)	(0.051)	(0.051)	(0.051)
Whether ethnic minorities	-0.113	-2.037*	-0.095	-0.095	-0.095	-0.095
	(0.098)	(1.138)	(0.061)	(0.061)	(0.061)	(0.061)
Whether religious believer	0.192**	1.688*	0.167**	0.167**	0.167**	0.167**
	(0.079)	(0.906)	(0.078)	(0.078)	(0.078)	(0.078)
Whether CPC member	0.148**	2.539***	-0.119***	-0.119***	-0.119***	-0.119***
	(0.063)	(0.780)	(0.044)	(0.044)	(0.044)	(0.044)
Weekly working hours	0.017***	0.196***	0.007*	0.007*	0.007*	0.007*
	(0.001)	(0.013)	(0.003)	(0.003)	(0.003)	(0.003)
Whether having pension	0.255***	3.142***	0.034	0.034	0.034	0.034
	(0.062)	(0.696)	(0.056)	(0.056)	(0.056)	(0.056)
Whether having medical insurance	0.153	2.187*	0.063	0.063	0.063	0.063
	(0.110)	(1.222)	(0.071)	(0.071)	(0.071)	(0.071)
Whether married	0.340***	4.905***	-0.113**	-0.113**	-0.113**	-0.113**
	(0.068)	(0.769)	(0.055)	(0.055)	(0.055)	(0.055)
Family size	-0.047***	-0.513***	-0.016	-0.016	-0.016	-0.016
	(0.017)	(0.194)	(0.014)	(0.014)	(0.014)	(0.014)
Number of children	-0.066**	-0.683**	-0.018	-0.018	-0.018	-0.018
	(0.028)	(0.318)	(0.023)	(0.023)	(0.023)	(0.023)
Number of houses	0.105***	2.440***	-0.066*	-0.066*	-0.066*	-0.066*
	(0.037)	(0.472)	(0.037)	(0.037)	(0.037)	(0.037)
Socio-economic status	0.268***	4.650***	-0.210***	-0.210***	-0.210***	-0.210***
	(0.047)	(0.537)	(0.039)	(0.039)	(0.039)	(0.039)
Year dummy	Yes	Yes	Yes	Yes	Yes	Yes

Province dummies	Yes	Yes	Yes	Yes	Yes	Yes
Constant	4.550***	28.970***	6.783***	6.783***	6.783***	6.783***
	(0.378)	(4.332)	(1.794)	(1.794)	(1.794)	(1.794)
Observations	11655	11655	11655	11655	11655	11655

Supplementary Figure 1. Placebo Test Results

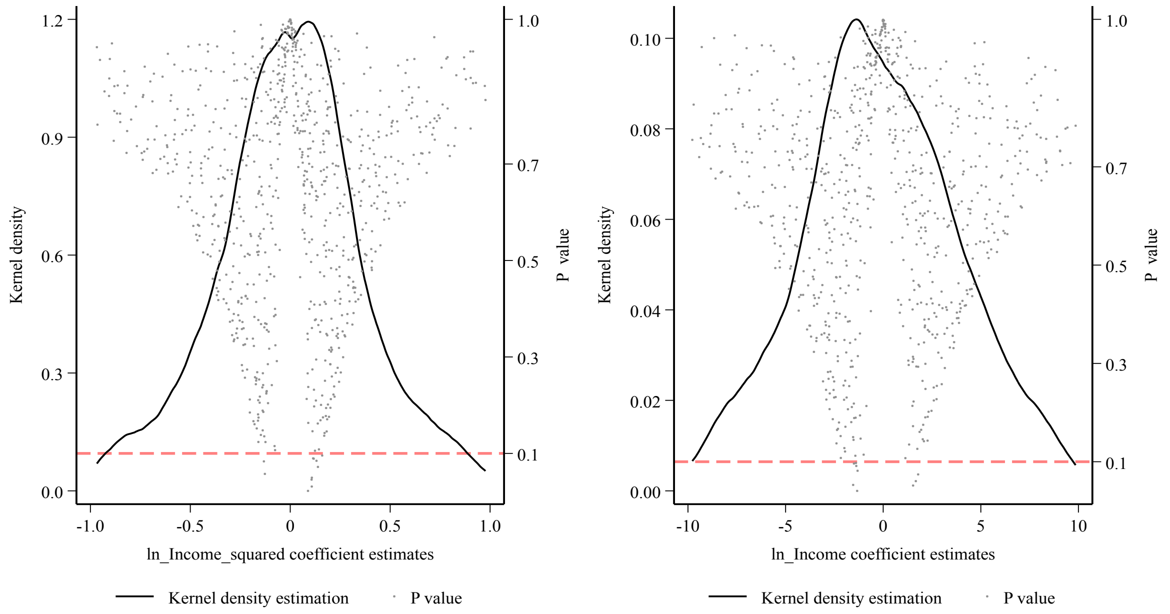

Supplementary Description of the Dataset

This paper uses a nationally representative observational dataset, which is the Chinese General Social Survey (CGSS). CGSS is in the world General Social Survey family, jointly carried out by Renmin University of China and Hong Kong University of Science and Technology. The sampling of CGSS is based on the multi-stage stratified design. The sampling stages are as follows: (1) PSUs are county-level units and there are 2762 PSUs in the sampling frame; (2) SSUs are community-level units (villages [cun] and neighborhood committees [ju wei hui]); (3) in selected SSU, 25 households (TSUs) are sampled with PPS method; (4) one eligible person aged 18-above is selected from each sampled household to serve as the representative. There are 43 Municipalities directly under the Chinese central government, provincial capital cities, and vice provincial cities in China. Comprehensive ranking by GDP, FDI and Education Level to these cities, the top 5 is Beijing, Shanghai, Tianjin, Guangzhou, and Shenzhen. CGSS treats these 5 cities as self-representative stratum. This stratum consists of 67 PSUs. The rest 2695 PSUs are comprehensively ranked with GDP per capital, urbanization rate, and population density and then are equally classified into 50 strata. Within each stratum, 2 PSUs will be selected with PPS method. In each selected PSU, 4 communities are sampled with PPS method. There are 80 communities in self-representative stratum and 400 communities in the rest 50 strata.

The national Survey Research Center at Renmin University of China (NSRC) organized Chinese Social Survey Network (CSSN), including 49 universities and provincial social science academies. Members of CSSN undertake the survey of the CGSS in their own provinces. The CGSS uses Computer Aided Personal Interviewing, and the average interview time is about one and a half hours. The CGSS has a set of strict quality control procedures, which cover pre-fieldwork, in-fieldwork, and after-fieldwork states. In pre-fieldwork stage, all supervisors must receive 40 hours training and finish 4 experimental interviews; all interviewers must receive 25 hours training and finish 3 experimental interviews. In in-fieldwork stage, all interviewers will be accompanied to interview by supervisor at least once. And the finished questionnaires must be 100% on site check and supervisors must do 40% the second day back interviewing. And in post-fieldwork stage, all interviews must 100% mail back interview and 40% telephone interview. In data input and coding stage, the data must be double input and double coding and there are several supervisors to check the double input and coding validation process.

The CGSS aims to collect quantitative data about (1) measures of social structure, its stability and change, (2) measures of quality of life, objective and subjective, and (3) measures of underlying mechanisms linking social structure and quality of life. The questionnaire of CGSS composes three kinds of modules: core module, topic module (rotation module), and additional module. The core module is annually repeated, which includes 152 variables. There are two kinds of the CGSS core module variables. One is the standard background variables, which include 71 variables. Another is the variables to trace social change trends. They are the rest 81 variables. The core module of the CGSS has 11 dimensions: Social demographic, health, lifestyle, migration, social attitude, class identity, political attitude and behavior, cognitive ability, labor market participation, social welfare, and family. The topic modules will be rotated every five years. There are one or two topic modules in the annual questionnaire. The topic modules aim to address important social issues. The additional modules include EASS module, ISSP module, and other ad hoc one-time modules. Some proposed topic modules also might be used as one-time additional modules. Questions in core module and topic modules will be asked to all respondents. Questions in additional modules only have one-third or a half chance to be asked.

Above information is taken from http://cgss.ruc.edu.cn/English/Home.htm.

Supplementary Description of the Automation Index

The automation index is calculated depending on respondents' occupation. This index is constructed by Autor and Dorn (2013) and has been the most commonly used indicator to characterize automation's impact (Acemoglu and Restrepo, 2020). The index is measured using the occupational characteristics of employment, from the US Department of Labor's *Dictionary of Occupational Titles* (DOT). A certain occupation involves multiple tasks at different levels of intensity, including the routine, abstract and manual tasks. It has been proved by the theoretical analysis in Autor and Dorn (2013) as well as other studies (e.g., Hubmer and Restrepo, 2022) that the higher the routine intensity and the lower the manual and abstract intensity of the occupation, the higher the extent of replacement by automation, thus the greater the impacts on labor income (Hubmer and Restrepo, 2022). Therefore, Autor and Dorn (2013) create a summary measure to characterize automation's impact on different occupations, calculated as

$$Automation_k = \ln\left(T_k^{Routine}\right) - \ln\left(T_k^{Manual}\right) - \ln\left(T_k^{Abstract}\right)$$

where $T_k^{Routine}$, T_k^{Manual}, and $T_k^{Abstract}$ are, respectively, the routine, manual, and abstract task inputs in each occupation k in *Dictionary of Occupational Titles* (DOT). Positive calculated measure would mean that the impact of artificial intelligence is to replace workers in the occupation and reduce income. This measure ranges from -6.190 to 4.235. According to this index, the $Automation$ indicator of those in sales and service elementary occupations, laborers in mining, construction, manufacturing and transport, and personal and protective service workers are equal or close to 4.235. This means that people's jobs in these occupations are replaced by automation to a larger extent, and consequently their income are more negatively affected by the exogenous AI technological progress. On the contrary, corporate managers, physical, mathematical and engineering professionals, life science and health professionals and other professionals' $Automation$ measure are equal or close to the minimum of -6.190. This means that those in above occupations are complementary to automation applications, and their income is positively affected by automation technology.

Supplementary Comparison Between Existing Literature and This Paper

No.	Title of the Paper	Publication Informatio n	Variables	Estimates	Contributio n of this paper	Reasons for the difference between the existing research and this paper
1	The relationship between income and subjective well-being: Relative or absolute?	Diener et al. (1993)	Dependent variable: Well-being<br><br>Independent variable: Income	Linear correlation: positive	Revised this conclusion	The quadratic term of income is not considered.
2	Influences and consequences of well-being among Australian young people: 1980- 1995	Marks and Fleming (1999)	Dependent variable: Income<br><br>Independent variable: Well-being	Linear correlation: positive	Revised this conclusion	The quadratic term of income is not considered.
3	Income and Happiness: Towards a Unified Theory	Easterlin (2001)	Theoretical analysis: X-axis: Income Y-axis: Subject Well-Being<br><br>Subject Well-Being as a function of income and aspiration level.	Linear correlation: positive.	Revised this conclusion	The quadratic term of income is not considered.

4	Dispositional Affect and Job Outcomes	Diener et al. (2002)	Dependent variable: Current Income<br><br>Independent variable: Parental Income<br><br>After dividing into 5 groups according to cheerfulness, the correlation between cheerfulness and current income is presented	U-shaped correlation: Current income first increases steeply as cheerfulness increases, levels off, and then decreases slightly.	Revised this conclusion	We use different variables as independent and dependent variables. Meanwhile, it didn't consider the quadratic term of income.
5	Does happiness pay? An exploration based on panel data from Russia	Graham et al. (2004)	Dependent variable: Income<br><br>Independent variable: Residual happiness	Linear correlation: positive.	Revised this conclusion	The quadratic term of income is not considered.
6	Socioeconomic status, depression disparities, and financial strain: what lies behind the income-depression relationship?	Zimmerman and Katon (2005)	Dependent variable: Depression (CESD)<br><br>Independent variable: Current income	Not significant	Rejected this conclusion	The quadratic term of income is not considered.

7	The relationship between pay and job satisfaction: A meta-analysis of the literature	Judge et al. (2010)	Dependent variable: Pay level<br><br>Independent variable: Job satisfaction, pay satisfaction	Linear correlation: positive	Revised this conclusion	The quadratic term of income is not considered.
8	Poverty and common mental disorders in low and middle income countries: A systematic review.	Lund et al. (2010)	This is a systematic review to explore poverty and common mental disorders.	Linear correlation: positive	Revised this conclusion	The quadratic term of income is not considered.
9	Buying happiness in an unequal world: Rank of income more strongly predicts well-being in more unequal countries.	Sareen (2011)	Dependent variable: Well-being<br><br>Independent variable: Income rank	Linear correlation: positive	Revised this conclusion	The quadratic term of income is not considered.
10	Population health status in China: EQ-5D results, by age, sex and socio-economic status, from the National Health	Sun et al. (2011)	Dependent variable: Mental health (Anxiety, depression)<br><br>Independent variable:	Linear correlation: negative	Revised this conclusion	The quadratic term of income is not considered.

	Services Survey 2008.		Income groups			
11	Estimating the influence of life satisfaction and positive affect on later income using sibling fixed effects	De Neve and Oswald (2012)	Dependent variable: Log income<br><br>Independent variable: Subjective well-being	Linear correlation: positive	Revised this conclusion	The quadratic term of income is not considered.
12	World Happiness Report 2012	Helliwell et al. (2012)	1. Empirical Analysis: Dependent variable: Life satisfaction (monthly). Independent variable: Log of income.<br><br>2. Theoretical analysis on the relationship between well-being and income	1. Linear correlation: positive<br>2. U-shaped: diminishing marginal utility of income	Revised this conclusion	1. The quadratic term of income is not considered.<br>2. There is no empirical analysis of the theory leading to diminishing margins.
13	How does money influence health?	Benzeval et al. (2014)	This research had reviewed theories from 272 wide-ranging papers, most of which examined the complex interactions between people's income and their health	Linear correlation: positive	Revised this conclusion	The quadratic term of income is not considered.

14	Organizational downsizing and depressive symptoms in the European recession: the experience of workers in France, Hungary, Sweden and the United Kingdom.	Brenner et al. (2014)	Dependent variables: Depressive symptoms<br><br>Independent variable: Unemployme nt	Linear correlation: positive	Revised this conclusion	The quadratic term of income is not considered.
15	Poverty and mental health in Indonesia.	Tampubolon and Hanandita (2014)	Dependent variables: Depressive symptoms (CES-D)<br><br>Independent variable: Poverty (individual social capital)	Linear correlation: positive	Revised this conclusion	The quadratic term of income is not considered.
16	Does money in adulthood affect adult outcomes.	Cooper and Stewart (2015)	This is a systematic review focuses on the relationship between income and adult outcomes which include subjective well-being.	Linear correlation: positive	Revised this conclusion	The quadratic term of income is not considered.
17	When does money matter most? Examining the association	Cheung and Lucas (2015)	Dependent variables: Life satisfaction	Linear correlation: positive	Revised this conclusion	The quadratic term of income is not considered.

	between income and life satisfaction over the life course.		Independent variable: Annual income			
18	Negative socioeconomic changes and mental disorders: a longitudinal study.	Barbaglia et al. (2015)	Dependent variables: Risk of mental disorder<br><br>Independent variable: Household income	Linear correlation: negative	Revised this conclusion	The quadratic term of income is not considered.
19	How financial hardship is associated with the onset of mental health problems over time.	Kiely et al. (2015)	Dependent variables: Mental health problems (MHI-5)<br><br>Independent variable: Current income	Linear correlation: positive	Revised this conclusion	The quadratic term of income is not considered.
20	Subjective wellbeing of Chinese people: A multifaceted view.	Bian et al.(2015)	Dependent variables: SWB<br><br>Independent variable: Ln (annual household income)	Linear correlation: positive	Revised this conclusion	The quadratic term of income is not considered.

21	Negative emotions, income, and welfare: Causal estimates from the PSID.	Clingingsmith (2016)	Dependent variables: Negative emotions (SD)<br><br>Independent variable: Family income	Linear correlation: positive	Revised this conclusion	The quadratic term of income is not considered.
22	Effects of a large-scale unconditional cash transfer program on mental health outcomes of young people in Kenya.	Kilburn et al. (2016)	Dependent variables: Depressive symptoms<br><br>Independent variable: Cash transfer	Linear correlation: negative	Revised this conclusion	The quadratic term of income is not considered.
23	Top incomes and human well-being: Evidence from the Gallup World Poll	Powdthavee et al. (2017)	Dependent variables: Positive emotional experience (enjoyment, happiness, well-rested, smile), negative emotional experience (worry, stress, anger, sadness)<br><br>Independent variable: Share of taxable income held	Linear correlation: positive (Happiness, stress, anger)<br><br>Negative (Enjoyment, rested, smile, worry, sadness)	Revised this conclusion	The quadratic term of income is not considered.

			by the top 1 percent in year t-1			
24	Do minimum wage increases influence worker health?	Horn et al. (2017)	Dependent variable: Mental health<br><br>Independent variable: Minimum wage	Linear correlation: positive	Revised this conclusion	The quadratic term of income is not considered.
25	The effect of income on subjective well-being evidence from the 2008 economic stimulus tax rebates.	Lachowska (2017)	Dependent variable: Subjective well-being<br><br>Independent variable: Income	Linear correlation: positive	Revised this conclusion	The quadratic term of income is not considered.
26	Financial circumstances, mastery, and mental health: Taking unobserved time-stable influences into account.	Koltai et al. (2018)	Dependent variable: Distress<br><br>Independent variable: Financial strain index	Linear correlation: positive	Revised this conclusion	The quadratic term of income is not considered.
27	Simultaneous social causation and social drift: Longitudinal analysis of depression and poverty in South Africa.	Lund and Cois (2018)	Dependent variable: Depression (CESD-10 scores)<br><br>Independent variable: Individual	Linear correlation: negative	Revised this conclusion	The quadratic term of income is not considered.

			asset index, household asset index.			
28	Paying for happiness: Experimental results from a large cash transfer program in Malawi.	Kilburn et al. (2018)	Dependent variable: Subjective well-being<br><br>Independent variable: Positive income shock (cash transfer)	Linear correlation: negative	Revised this conclusion	The quadratic term of income is not considered.
29	Does money relieve depression? Evidence from social pension expansions in China.	Chen et al. (2019)	Dependent variable: Depression (Measured by total score of CES-D)<br><br>Independent variable: Pension enrollment	Linear correlation: negative	Revised this conclusion	The quadratic term of income is not considered.
30	Socioeconomic position and depression in South African adults with long-term health conditions: a longitudinal study of causal pathways.	Elwell-Sutton et al. (2019)	Dependent variable: Depression score (Measured by CESD-10 score)<br><br>Independent variable: Income	Linear correlation: positive	Revised this conclusion	The quadratic term of income is not considered.

31	Prevalence and socioeconomic impact of depressive disorders in India: multisite population-based cross-sectional study.	Arvind et al. (2019)	Dependent variable: Prevalence of depressive disorders<br><br>Independent variable: Income quintile	Linear correlation: negative	Revised this conclusion	The quadratic term of income is not considered.
32	Poverty, depression, and anxiety: Causal evidence and mechanisms.	Ridley et al. (2020)	This is a systematic review explored poverty and mental illness such as depression and anxiety.	Linear correlation: positive	Revised this conclusion	Maybe the quadratic term of income is not considered.
33	Buying happiness in an unequal world: Rank of income more strongly predicts well-being in more unequal countries	Macchia et al. (2020)	Dependent variable: Well-being<br><br>Independent variable: Income rank	Linear correlation: positive	Revised this conclusion	The quadratic term of income is not considered.
34	Impact of COVID-19 on economic well-being and quality of life of the Vietnamese during the national social distancing.	Tran et al. (2020)	Dependent variables: Health-related quality of life (EuroQoL 5 Dimensions 5 Levels, EQ-5D-5L)<br><br>Independent variable:	Linear correlation: positive	Revised this conclusion	The quadratic term of income is not considered.

			Household income			
35	Association of negative financial shocks during the Great Recession with depressive symptoms and substance use in the USA: the CARDIA study.	Swift et al. (2020)	Dependent variables: Depressive symptoms (Change in CESD score between 2005 and 2010)<br><br>Independent variable: Income drop	Linear correlation: positive	Revised this conclusion	The quadratic term of income is not considered.
36	Spousal relative income and male psychological distress.	Syrda (2020)	Dependent variables: Male psychological distress<br><br>Independent variable: Wife's relative income	U-shaped correlation: Controlling for total household income, predicted male psychologica l distress reaches a minimum at a point where wives make 40% of total household income and proceeds to increase, to reach highest level when men are entirely economicall y dependent on their wives.	Revised this conclusion	We use different format of income. The existing research considered the situation between spouse, while our paper explores the relationship one the individuals level.

37	Change in work-related income following the uptake of treatment for mental disorders among young migrant and non-migrant women in Norway: a national register study.	Hynek et al. (2021)	Dependent variables: Uptake of OPMH treatment (a proxy for mental disorder)<br><br>Independent variable: Income	Linear correlation: negative	Revised this conclusion	The quadratic term of income is not considered.
38	Association Between Household Income and Self-Perceived Health Status and Poor Mental and Physical Health Among Cancer Survivors.	Su et al. (2021)	Dependent variables: HRQoL<br><br>Independent variable: Income	Linear correlation: positive	Revised this conclusion	The quadratic term of income is not considered.
39	Do social pension schemes promote the mental health of rural middle-aged and old residents? Evidence from China.	Pan et al. (2021)	Dependent variables: Level of depressive symptoms, probability of depression<br><br>Independent variable: Enrollment in NRSP (dummy variable)	Linear correlation: negative	Revised this conclusion	The quadratic term of income is not considered.

40	World Happiness Report 2021	Helliwell et al. (2021)	Dependent variable: Life Satisfaction<br><br>Independent variables:<br>- Stopped work, furloughed, no income loss<br>- Stopped work, furloughed, income loss<br>- Stopped work, not furloughed, income loss	Linear correlation: negative	Revised this conclusion	The quadratic term of income is not considered.
41	How do changes in individual or household income impact on mental health for working-age adults? A systematic review.	Thomson et al. (2021)	This paper searched MEDLINE, Embase, Web of Science, PsycINFO, ASSIA, EconLit and RePEc for randomized controlled trials (RCTs) and quantitative non-randomized studies, explore the relationship between mental health and	Linear correlation: positive	Revised this conclusion	The quadratic term of income is not considered.

			household income.			
42	Association among income loss, financial strain and depressive symptoms during covid-19: evidence from two longitudinal studies.	Hertz-Palmor et al. (2021)	Dependent variable: Depression, anxiety<br><br>Independent variable: Income loss	Linear correlation: positive	Revised this conclusion	The quadratic term of income is not considered.
43	The impact of income on mental health	Shields and Filip (2022)	Dependent variable: Mental health<br><br>Independent variable: Income	Linear correlation: positive	Revised this conclusion	The quadratic term of income is not considered.
44	How do income changes impact on mental health and wellbeing for working-age adults?	Thomson et al. (2022)	Dependent variable: Mental health and well-being<br><br>Independent variable: Individual and household income	Linear correlation: positive	Revised this conclusion	The quadratic term of income is not considered.
45	Prevalence and determinants of probable depression and anxiety during the	Hajek et al. (2022)	Dependent variable: Probable depression, probable anxiety	Linear correlation: positive	Revised this conclusion	The quadratic term of income is not considered.

	COVID-19 pandemic in seven countries: Longitudinal evidence from the European COvid Survey (ECOS).		Independent variable: Income with great difficulty			
